# Supplementary material for: Measuring Spatial and Temporal PM2.5 Variations in Sacramento, California, Communities Using a Network of Low-Cost Sensors
Source: Sensors (Basel). 2019 Oct 29;19(21):4701. doi: 10.3390/s19214701 (PMC6864658; doi:10.3390/s19214701)
Supplement: Supplementary file 1 [file sensors-19-04701-s001.pdf]

Measuring Spatial and Temporal $PM_{2.5}$ Variations in Sacramento, California, Communities Using a Network of Low-Cost Sensors

Anondo Mukherjee [1,2], Steven G. Brown [1,*], Michael C. McCarthy [1], Nathan R. Pavlovic [1], Levi G. Stanton [1], Janice Lam Snyder [3], Stephen D'Andrea [3] and Hilary R. Hafner [1]

[1] Sonoma Technology, 1450 N. McDowell Blvd., Suite 200, Petaluma, CA 94954, USA; amukherjee@sonomatech.com (A.M.); mmccarthy@sonomatech.com (M.C.M.); npavlovic@sonomatech.com (N.R.P.); lstanton@sonomatech.com (L.G.S.); hilary@sonomatech.com (H.R.H.)
[2] Department of Atmospheric and Oceanic Sciences, University of Colorado Boulder, Boulder, CO 80309, USA
[3] Sacramento Metropolitan Air Quality Management District (SMAQMD), Sacramento, CA 95814, USA; jlam@airquality.org (J.L.S.); sdandrea@airquality.org (S.D.)
* Corresponding author: sbrown@sonomatech.com; Tel.: +1-707-665-9900; Fax: +1-707-665-9800

Supplementary Material

Table S1. The change in measurement from the pre- and post-study periods over a range of measurement values, using the linear regressions from the pre- and post-study periods presented in Table 1.

AirBeam	Change in 5ug/m3	Change in 10 ug/m3	Change in 20 ug/m3	Change in 30 ug/m3	Change in 40 ug/m3	Change in 50 ug/m3	% Change in 5 ug/m3	% Change in 10 ug/m3	% Change in 20 ug/m3	% Change in 30 ug/m3	% Change in 40 ug/m3	% Change in 50 ug/m3
13th Ave	−0.03	−0.29	−0.81	−1.33	−1.85	−2.37	−0.58	−2.89	−4.05	−4.44	−4.63	−4.75
24th Ave	−0.21	−0.36	−0.66	−0.96	−1.26	−1.56	−4.29	−3.64	−3.32	−3.21	−3.16	−3.13
64th St	0.61	0.39	−0.05	−0.49	−0.93	−1.37	12.27	3.93	−0.24	−1.63	−2.33	−2.74
ARB T St 2	0.22	0.72	1.72	2.72	3.73	4.73	4.31	7.17	8.60	9.08	9.32	9.46
ARB T St 3	0.75	0.39	−0.32	−1.04	−1.76	−2.47	15.00	3.92	−1.62	−3.47	−4.39	−4.95
Alderwood	1.16	0.71	−0.19	−1.10	−2.00	−2.90	23.26	7.11	−0.96	−3.66	−5.00	−5.81
Coroval	0.30	−0.09	−0.87	−1.65	−2.43	−3.21	6.08	−0.86	−4.33	−5.49	−6.07	−6.42
Del Paso 2	1.22	0.71	−0.31	−1.34	−2.36	−3.38	24.31	7.05	−1.57	−4.45	−5.89	−6.75
Del Paso 3	−0.13	−0.45	−1.07	−1.69	−2.32	−2.94	−2.66	−4.45	−5.35	−5.64	−5.79	−5.88
Darwin St	0.53	0.28	−0.22	−0.72	−1.22	−1.72	10.60	2.80	−1.09	−2.39	−3.04	−3.43
Henrietta Dr	−0.79	−1.13	−1.79	−2.45	−3.12	−3.78	−15.85	−11.25	−8.95	−8.18	−7.80	−7.57
Socorro Way	0.76	0.53	0.08	−0.38	−0.84	−1.30	15.28	5.35	0.38	−1.27	−2.10	−2.59
Tristan Cir	−1.87	−1.75	−1.51	−1.27	−1.04	−0.80	−37.32	−17.48	−7.56	−4.25	−2.60	−1.60
Wyman	0.01	0.12	0.35	0.57	0.80	1.02	0.26	1.25	1.75	1.91	1.99	2.04
79thSt	0.99	1.56	2.69	3.83	4.96	6.10	19.80	15.57	13.46	12.76	12.40	12.19
ARB T St	−0.86	−0.56	0.03	0.62	1.20	1.79	−17.15	−5.63	0.13	2.05	3.01	3.59
Del Paso	−0.03	0.26	0.86	1.45	2.04	2.64	−0.69	2.63	4.28	4.84	5.11	5.28
Hermosa St	−0.01	0.14	0.44	0.75	1.05	1.35	−0.16	1.43	2.22	2.49	2.62	2.70
T St Tier 3	−2.11	−1.59	−0.54	0.52	1.57	2.62	−42.25	−15.87	−2.68	1.72	3.92	5.24

Table S2. Matrix showing the p-value results of the Pairwise Wilcoxon Rank test for PM measurements in EJ vs. non-EJ sites; EJ sites are in the first column, and non-EJ sites are in the top row. A p-value equal to 1 indicates that no statistical difference in the mean between the two sites can be determined. A p-value less than 0.05 indicates that a difference in the mean is significant at the 95% confidence level.

	**Alderwood**	**ARB T St**	**Del Paso Manor**	**T St Tier 3**	**Wyman**	**13th Ave**	**64th St**	**79th St**
Coroval	1	1	0.14	1	0.66	1	1	0.001
Darwin St	1	0.68	1	1	1	1	1	1
Henrietta Dr	1	1	1	1	1	1	1	0.06
Hermosa St	1	0.74	1	1	1	1	1	1
Socorro Way	1	1	1	1	1	1	1	0.13
Tristan Cir	1	0.0005	1	0.45	1	1	1	1
24th Ave	1	1	1	1	1	1	1	0.04

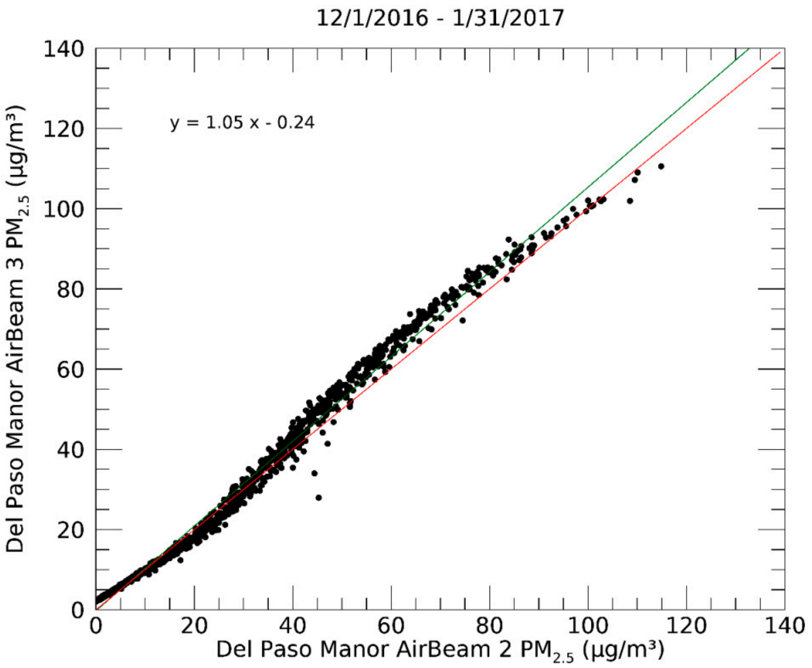

Figure S1. Scatterplot showing the correlation between two collocated AirBeams at Del Paso Manor during the study period. Linear regression, y = 1.05 x − 0.24 shown in green. 1:1 line shown in red.

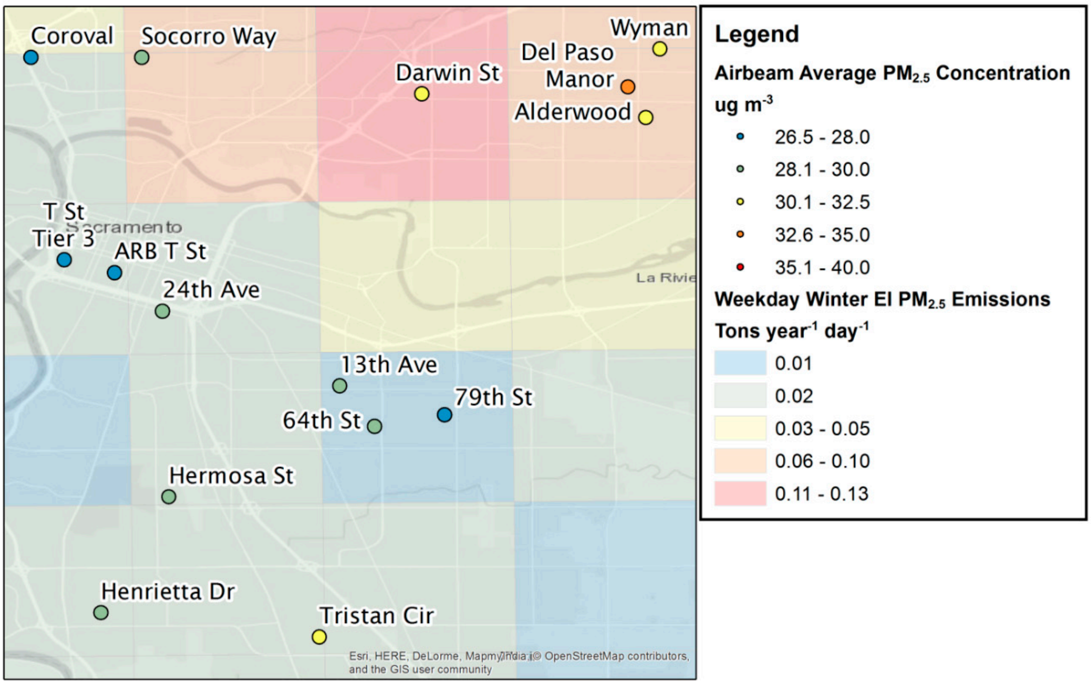

Figure S2. Map of 4-by-4 km gridded winter weekday $PM_{2.5}$ emissions and average AirBeam $PM_{2.5}$ concentrations from the December 2016 to January 2017 period.

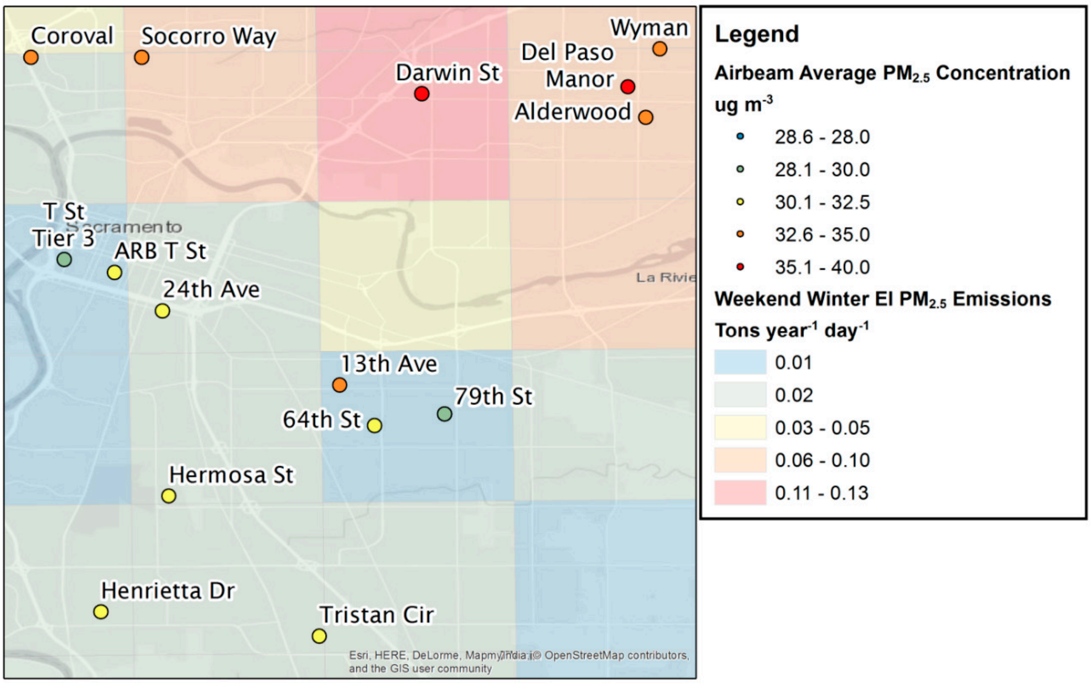

Figure S3. Map of 4-by-4 km gridded winter weekend $PM_{2.5}$ emissions and average AirBeam $PM_{2.5}$ concentrations from the December 2016 to January 2017 period.
